# Frequency of quinolone resistance genes among extended-spectrum β-lactamase (ESBL)-producing *Escherichia coli* strains isolated from urinary tract infections

**DOI:** 10.1186/s41182-019-0147-8

**Published:** 2019-03-04

**Authors:** Ahmad FarajzadehSheikh, Hojat Veisi, Mojtaba Shahin, Muhammad Getso, Abbas Farahani

**Affiliations:** 1 Infectious and Tropical Diseases Research Center, Health Research Institute, Ahvaz Jundishapur University of Medical Sciences, Ahvaz, Iran; 2 Department of Microbiology, School of Medicine, Ahvaz Jundishapur University of Medical Sciences, Ahvaz, Iran; 3 Department of Microbiology, Arvand international Division, Ahvaz Jundishapur University of Medical Sciences, Ahvaz, Iran; 4 Department of Medical Microbiology and Parasitology, College of Health Sciences, Bayero University, PMB, Kano, 3011 Nigeria; 5 Infectious and Tropical Diseases Research Center, Hormozgan Health Institute, Hormozgan University of Medical Sciences, Bandar Abbas, Iran

**Keywords:** *E. coli*, UTIs, *Qnr*, ESBL

## Abstract

**Background:**

As an opportunistic pathogen, *Escherichia coli* (*E. coli*) is widely recognized as the main cause of nosocomial infections as well as some disorders especially those associated with urinary tract infections (UTIs). This study, therefore, sets out to determine the extent of antibiotic resistance to quinolones and to measure the frequency of *qnr* genes (A, B, and S) within extended-spectrum beta-lactamase (ESBL) and non-ESBL-producing strains of *E. coli* isolated from UTI-diagnosed patients as well as to investigate their antimicrobial susceptibility patterns for some selected antibiotics in southwest Iran.

**Methods:**

Two hundred *E. coli* strains were isolated from UTI-diagnosed patients, hospitalized in nine different wards of Ahvaz Golestan Hospital between November 2015 and March 2016. The isolates were confirmed through well-practiced phenotypical methods. Moreover, the antimicrobial susceptibility test was successfully performed using a disk diffusion method. ESBL production among the isolates was screened by double disk synergism test (DDST), and the *qnr* genes were identified using a multiplex PCR.

**Results:**

Out of the 200 samples collected, 167 isolates were confirmed to be *E. coli* strains. Maximum and minimum resistance were reported against nalidixic acid and chloramphenicol with 65.3% and 17.4%, respectively. Most of the isolates were resistant to all three types of quinolones studied in this research. Using multiplex PCR, the *qnr* genes were found in 100 (59.88%) strains (*qnr*A = 10, *qnr*B = 21, *qnr*S = 41, *qnr*B-S = 21, *qnr*B-A = 1, *qnr*A-S = 3, *qnr*A-B-S = 3), 58% of which was found among ESBL-producing isolates.

**Conclusions:**

Resistance to quinolones antibiotics was highest among ESBL-producing isolates harboring, especially *qnr*S among other determinants of the *qnr* gene. There is a need for sensitive antibiotic stewardship especially in hospitals of Ahvaz, Khuzestan province. Further research is needed to ascertain the gravity of quinolones resistance in Iran and to quickly act against its spread among other nosocomial pathogens.

## Background

As an opportunistic pathogen, *Escherichia coli (E. coli)* is regarded as the main cause of nosocomial infections, especially such medical disorders as UTIs (urinary tract infections), sepsis, aspiratory infections, and meningitis among neonates. The outbreak of plasmid-mediated quinolone resistance (PMQR) has steadily been increasing in recent years, leading to a decline in optimal therapeutic options [1].

Quinolones are a group of broad-spectrum antimicrobial agents that are helpful in the treatment of several infections caused by gram-negative bacteria, especially *E. coli.* However, over the past few years, the gram-negative bacteria have increasingly turned to be resistant to quinolones in Iran [1, 2].

Quinolones are a group of wide-spectrum antimicrobials that are often used to treat *E. coli* infections. Several genes and systems involved in quinolone resistance Enterobacteriaceae isolates*.* Quinolone resistance gene (*qnr*), a member of PMQR determinants, was initially discovered in a strain of *Klebsiella pneumoniae* (*K. pneumonia)*.

The first member of this family (*qnr*_s_) is *qnr*A that was discovered in the USA in 1998 [1, 3]. *Qnr* proteins are responsible for protecting the target enzymes including topoisomerase IV and DNA gyrase against quinolones such as ciprofloxacin and norfloxacin [4].

*Qnr* genes such as *qnr*A, *qnr*B, and *qnr*S are three types of the main classes of *qnr* determinants which have been identified in several members of Enterobacteriaceae family such as *E. coli* and *K. pneumonia* [3, 5, 6].

Over the past few years, researchers have discovered some other classes of the gene namely *qnr*C and *qnr*D [6, 7]. The *qnr* gene product is composed of pentapeptide repeat proteins which protect the strains against the effects of quinolone antibiotics. Moreover, *qnr* gene products may result in 8 to a 32-fold rise in minimum inhibitory concentration (MIC) against these groups of antibiotics, enabling these types of proteins to reduce the ciprofloxacin activity. Structural modification in its structure may follow antibiotic pressure, leading to resistance to quinolones thereafter [8].

The extent of quinolone resistance in clinical isolates such as *E. coli* is unexpectedly high in Iran, especially in those strains that produce ESBLs [1]. Moreover, ESBLs genes have often been reported as being co-associated with genes that encode PMQR (*qnr*, *aac* (6′)-Ib-cr and *qep*A.) and sometimes other families of ESBL genes (*blaSHV* and *blaTEM*) have been found located together with *qnr* genes on a special plasmid in a pathogen isolate [9, 10]. The *qnr* genes are widespread all around the world, especially in hospitalized patients.

This study, therefore, is set out to determine the extent of antibiotic resistance to quinolones and to measure the frequency of *qnr* genes (A, B, and S) within non-ESBL and ESBL-producing strains of *E. coli* isolated from UTI-diagnosed patients as well as to investigate their antimicrobial susceptibility patterns for some selected antibiotics in southwest Iran.

## Materials and methods

### Patients and bacterial strains

Two hundred bacterial isolates from the urine of UTI-diagnosed patients were collected from the clinical laboratory of Golestan Hospital (affiliated to the Jundishapur Medical University of Ahvaz, Khuzestan province, Iran). The isolates were from both male and female outpatients and inpatients diagnosed with UTI who presented at different wards of Golestan Hospital such as urology, nephrology, ICU, internal, pediatric, transplant, psychology, and outpatient department (OPD) from November 2015 to March 2016. The isolates were then transported to the Department of Medical Microbiology in the Jundishapur University of Ahvaz for further processing and analysis as outlined below. The cases were divided into nine age groups (1, 0–10; 2, 11–20; 3, 21–30; 4, 31–40; 5, 41–50; 6, 51–60; 7, 61–70; 8, 71–80; 9, 81–90). Out of the 200 clinical bacterial isolates collected from hospital laboratory, 167 isolates were confirmed as bacilli with the ability to grow in MacConkey agar and fermented lactose (overnight), tested negative with Gram’s staining, oxidase test, triple sugar iron agar, SH_2_-indol-motility, Voges-Proskauer, urease test, and citrate utilization test but positive with motility, indole, and *Methyl Red* tests [11]. All 167 isolates were confirmed using the API20E identification kit (bioMerieux SA, Marcy l’Etoile, France) to *E. coli* [12].

### Antimicrobial susceptibility pattern

The antimicrobial susceptibility test was performed using the disk diffusion method, in Muller-Hinton agar (MHA, Merck, Germany) plate. The antibiotics disc tested based on the minimal growth inhibitory zone diameter were nalidixic acid (NA = 30 μg), ciprofloxacin (Cip = 5 μg), ofloxacin (OF = 5 μg), chloramphenicol (CL = 30 μg), cefotaxime (30 μg), ceftazidime (30 μg) and aztreonam (AZT = 30 μg) (Mast Group, Bootle, UK) [13]. The results were interpreted as resistant, intermediate or sensitive, in accordance with the guidelines of the CLSI (2015) and the manufacturer protocols [4]. The strains used for quality control were *E. coli* ATCC 25922 and *Enterococcus fecalis* ATCC 29212.

### ESBLs screening test

A DDST method was used to screen for ESBLs production among the tested isolates. To all isolates that were identified as resistant strains to ceftazidime or cefotaxime (on MHA media), a double disk (containing clavulanic acid and ceftazidime or clavulanic acid and cefotaxime) was placed (near the single disk). If there was ≥ 5 mm extra zone diameter difference between the single disk and the double disk, after overnight incubation, the test strain was reported as ESBL positive. *K. pneumoniae* ATCC 700603 and *E. coli* ATCC 25922 were used as positive and negative control strains respectively, in accordance with CLSI guidelines.

### Detecting *qnr* (A, B, and S) genes by multiplex PCR technique

At first *E. coli* isolates were cultivated in Luria-Bertani broth at 37 °C overnight. At the second step, four to five colonies of the isolates were transferred to a microtube containing 1.5 ml DW (water), and finally, the bacterial DNA was extracted for molecular analysis [3] via boiling method. Based on the sequence arrangements of the target genes, six primers were used [14] to amplify the internal fragments of 580 bp, 264 bp, and 428 bp for *qnr*A, B, and S genes, respectively. Nucleotide sequence primers used for detection of *qnr* (A, B, and S) genes by the multiplex PCR method are presented in Table 1. The multiplex PCR was performed in a 50 μl reaction mixture, containing 2 μl of total DNA, 6 μl of the primers (1 μl for each), 22 μl of the DW, and 20 μl of the master kit (Sinaclon, made in Iran). Target fragments were amplified using a thermal cycler (Eppendorf, Germany) as follows: initial denaturation at 94 ^°^C for 4 min, 30 cycles of amplification consisting of denaturation at 94 ^°^C for 30 s, annealing at 53 ^°^C for 45 s, extension at 72 ^°^C for 30 s, and a final extension stage at 72 ^°^C for 6 min. The electrophoresis of the PCR products was performed on 2% agarose gel stained with ethidium bromide (0.5 μg/ml) as follows: 5 μl of PCR product was mixed with 1 μl of loading buffer, and then run at 110 V for 50 min in 1× TBE [tris 54 g(pH ± =8–8.3), 27.5 g boric acid, 20 ml EDTA] solution. All positive controls for *qnr*A, B, and S were obtained from Pastor Institute, Iran. The products of these genes (*qnr*A, B, and S) were sequenced (Macrogen Company, Korea) and confirmed in the NCBI database (http://blast.ncbi.nlm.nih.gov/).

**Table 1:** Primers for polymerase chain reaction of *qnr* (A, B, and S) genes

Primers^a^	Nucleotide sequences	Size	Reference
*qnr*A-F	5-AGAGGATTTCTCACGCCAGG-3	580 bp	[14]
*qnr*A-R	5-TGCCAGGCACAGATCTTGAC-3		
*qnr*B-F	5-GGMATHGAAATTCGCCACTG-3	264 bp	
*qnr*B-R	5-TTTGCYGYYCGCCAGTCGAA-3		
*qnr*S-F	5-GCAAGTTCATTGAACAGGGT-3	428 bp	
*qnr*S-R	5-TCTAAACCGTCGAGTTCGGCG-3		

^a^*F* sense primer, *R* antisense primer

### Statistical analysis

The statistical analysis was done using SPSS software (IBM, Chicago, IL, USA) version 20. For the purposes of this study, Fisher’s test and the non-parametric chi-squared test were performed and *P* value < 0.05 was considered statistically significant.

## Results

**Fig. 1:**
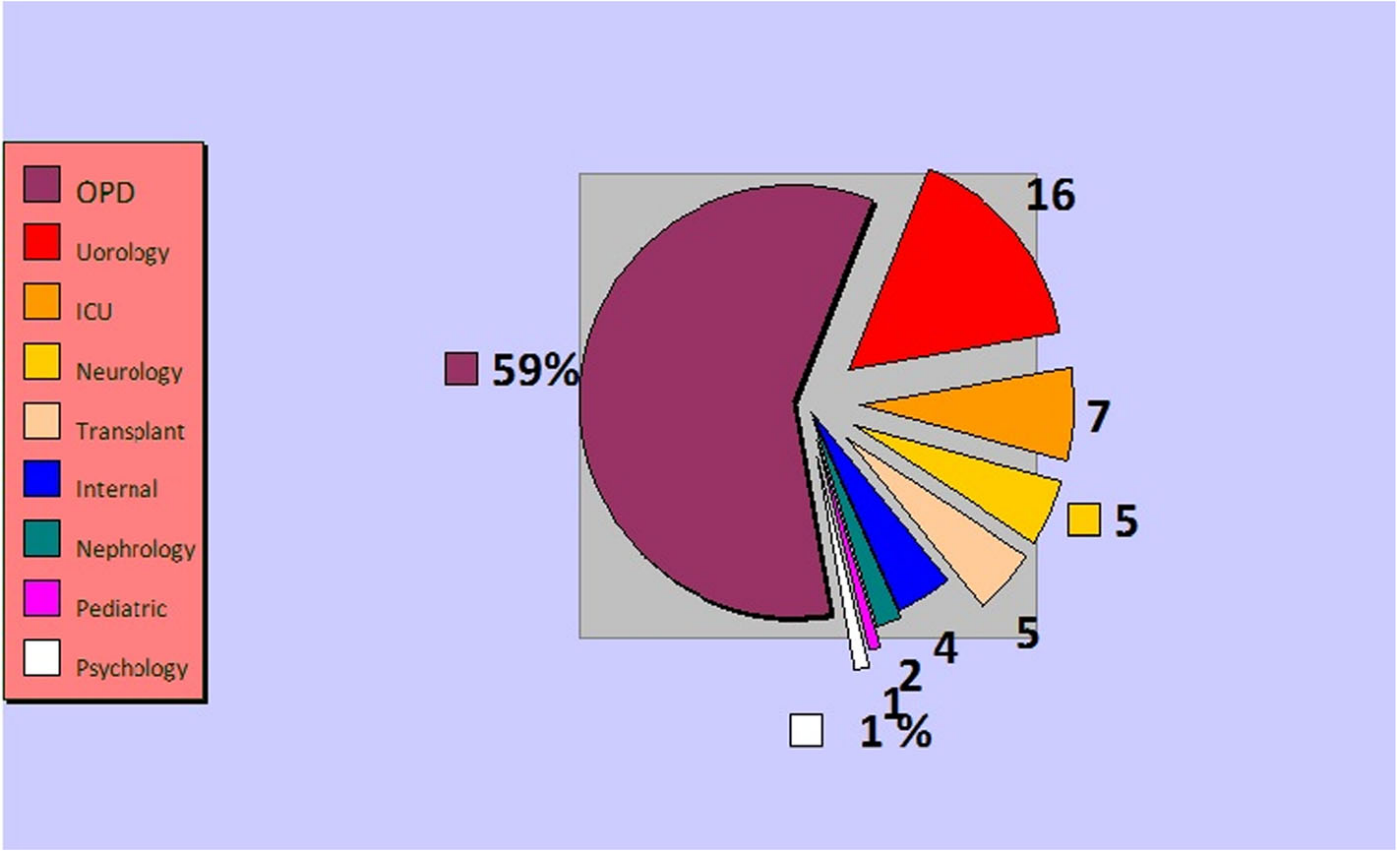
The percentage of *qnr* genes among *Escherichia coli* isolates in nine wards. ICU, intensive care unit; OPD, outpatient department

**Fig. 2:**
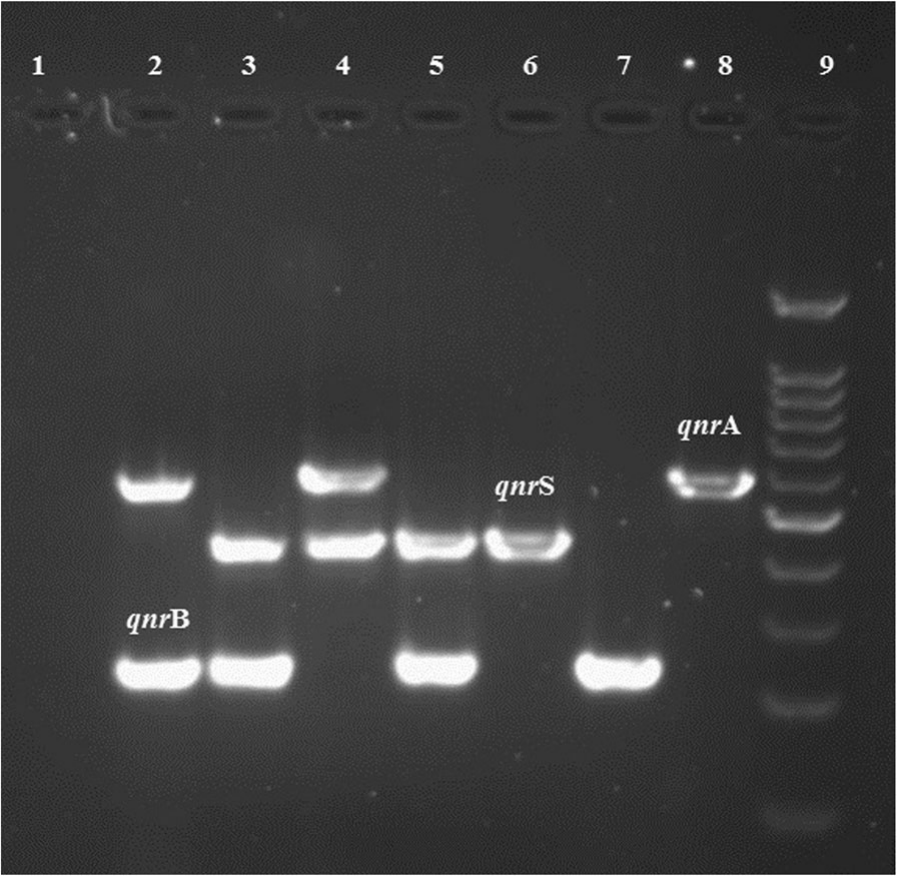
Agarose gel electrophoresis of *qnr*A (580 bp), *qnr*B (264 bp), and *qnr*S (428 bp) genes of multiplex PCR amplification products. Left to right lane: lane 1 negative control; lanes 6, 7, and 8 positive control for *qnr*S, *qnr*B, and *qnr*A, respectively; lane 2 *qnr*A and B; lane 3 *qnr*S and B; lane 4 *qnr*A and S; lane 9 100 bp DNA ladder

Out of the 200 urinary bacterial isolates collected from the hospital, a total of 167 isolates was confirmed as *E. coli*. Most of the isolates were gathered from OPD (59%) (Fig. 1). The total cases included 40.72% inpatients and 59.21% outpatients; 68.26% of whom were females and 31.74% were males. The majority (39%) of these patients belonged to the third (21–30 year) and forth (31–40 year) age groups. The results of the antibacterial susceptibility test revealed that most isolates were resistant to NA (65.3%), while least resistance was demonstrated against CL (17.4%) (Table 2). Resistance to NA (37.61%) and Cip (44.28%) was mostly seen among the third and the fourth (21–40 age) age groups. The highest percentage of resistance to NA (70.83%, 17/24) and Cip (66.66%, 16/24) was seen among the isolates collected via the urology ward. Interestingly, most of the isolates were resistant to all the three types of quinolone antibiotics tested upon (Table 2). The results of ESBLs screening revealed that 64.07% (107/167) of the tested *E. coli* strains were ESBL positive, out of which 65.91% were isolated from the outpatients and the rest from the inpatients. The majorities of ESBL- producing bacteria were isolated from females (66.35%) and were also gathered via OPD, whereas the least proportion of the isolates (1.19%) was collected from psychology wards. The *qnr* genes were successfully amplified by multiplex PCR technique (Fig. 2), and the results of this technique revealed that 100/167 (59.88%) of the isolates harbored the *qnr* genes with the following distributions: *qnr*A, *n* = 10; *qnr*B, *n* = 21; *qnr*S, *n* = 41; *qnr*B-S, n = 21; *qnr*B-A, *n* = 1; *qnr*A-S, *n* = 3; *qnr*A-B-S, *n* = 3 (Table 3).

**Table 2:** Antimicrobial-susceptibility for *Escherichia coli* isolates

Antibiotics (AB)	Susceptibility; no. (%) of isolates		
	Susceptible (S)	Intermediate (I)	Resistant (R)
Aztreonam (AZT)	76 (45.5)	22 (13.2)	69 (41.3)
Cefotaxime (CTX)	57 (34.1)	8 (4.8)	102 (61.1)
Ceftazidime (CAZ)	66 (39.5)	16 (9.6)	85 (50.9)
Ciprofloxacin (CIP)	67 (40.1)	13 (7.8)	87 (52.1)
Chloramphenicol (CL)	110 (65.9)	28 (16.8)	29 (17.4)
Nalidixic acid (NA)	53 (31.7)	5 (3)	109 (65.3)
Ofloxacin (OF)	75 (44.9)	4 (2.4)	88 (52.7)

**Table 3:** Frequency of different *qnr* genes, among ESBL pos, ESBL neg, and outpatients and inpatients

Qnr A,B, and S	ESBL		Patients	
	Positive	Negative	Outpatients	Inpatients
QnrA	6	4	8	2
QnrB	8	13	12	9
QnrS	30	11	22	19
QnrS-B	12	9	13	8
QnrA-S	0	3	2	1
QnrA-B	0	1	1	0
QnrA-B-S	2	1	1	2
Total isolates	58	42	59	41
	100		100	

*Abbreviations*: *ESBL pos* ESBL positive, *ESBL neg* ESBL negative

**Fig. 3:**
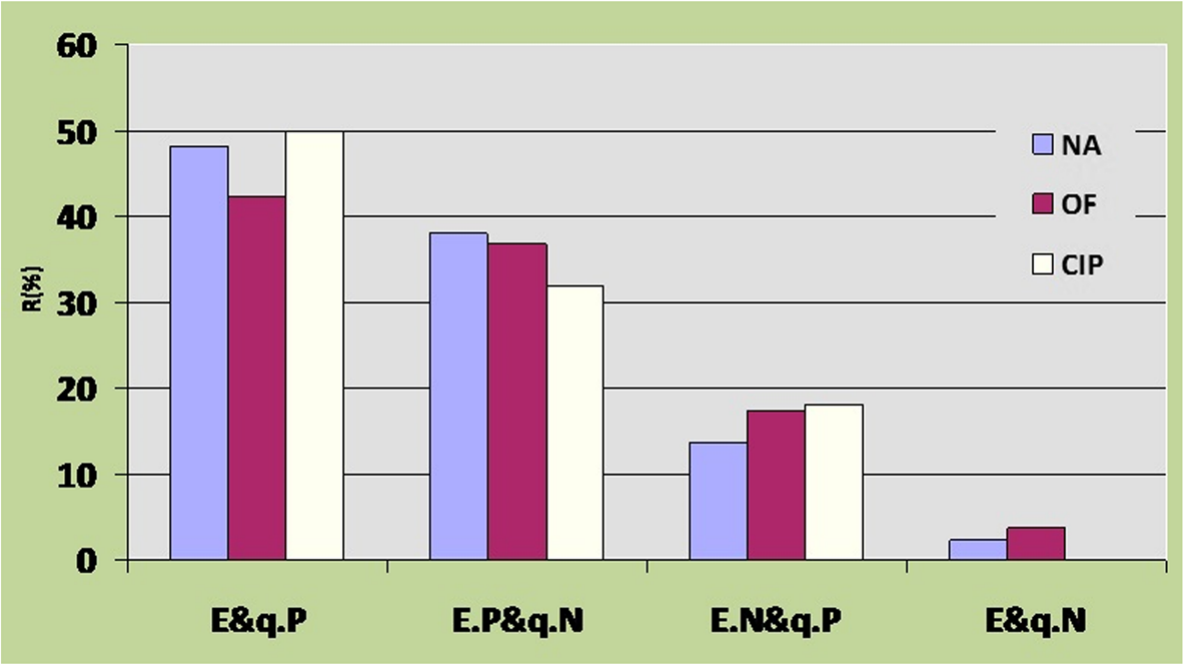
Quinolone resistance pattern of ESBL-producing *Escherichia coli* isolates harboring *qnr* genes. Left to right: E&q.P, ESBL and *qnr* positive; E.P&q.N, ESBL positive and *qnr* negative; E.N&q.P, ESBL negative and *qnr* positive; E&q.N, ESBL and *qnr* negative

Fifty-eight percent (58/100) of the *qnr* genes-carrying isolates were ESBL producers (Table 3). Among the ESBL and *qnr* positive strains, 9/58 (15.5%) were sensitive to all quinolone antibiotics (Fig. 3). The study also revealed that 65.85% of the 41 isolates carrying only *qnr*S gene were resistant to all the three types of quinolone antibiotics (NA, OF, Cip), suggesting that *qnr*S gene seems to result in more resistance to quinolones than *qnr*B and *qnr*A (Table 4). A significant correlation (*P*_value_ < 0.05) was observed between ciprofloxacin and ofloxacin among the strains harboring *qnr*S genes (Table 4). Moreover, in cases where a combination of *qnr*S and B was detected, the resistance to quinolones escalated to over 95% (Table 4). The relationship between ESBLs production, presence of *qnr* genes, and the quinolone resistance patterns among the tested strains is shown in Fig. 3.

**Table 4:** Percentage of resisting isolates to nalidixic acid, ofloxacin, and ciprofloxacin, among strains which have harbored just one type of *qnr* genes and/or *qnr* S and B

Antibiotics	*qnr* B	*qnr* A	*qnr* S	*qnr* S-B
	21 (no)	10 (no)	41 (no)	21 (no)
Nalidixic acid (%)	33.33	60.00	73.17	100
Ofloxacin (%)	28.57	50.00	70.73*	95.23
Ciprofloxacin (%)	28.57	50.00	68.29*	95.23
NA, OF, and CIP (%)	28.57	50.00	65.85	95.23

*Abbreviations*: *R* resistant, *no* number

*P value < 0.05

## Discussion

Over the last decades*, E. coli* has been recognized as one of the leading causes of nosocomial infections in the world. In principle, some genes located on the especial plasmids in such strains are regarded as being responsible for such problems [15, 16]. By developing some proteins, the genes (*qnrs*, ESBLs) build up resistance to antibiotics [17]. Though *qnr* genes and ESBLs are the most significantly resistant agents (against β-lactam and quinolone drugs), recently, the rate of resistance to other antibiotics has sharply risen [1, 17]. In our study, the majority (68.26%) of the UTI-diagnosed patients were women. This might be related to the anatomical position of women^**’**^s urethral tract closer to the anal canal; in addition, most of these patients belong to the third and fourth age groups (aged between 21and 40 years)—the most sexually active age group. Thus, it could be concluded that sex and age are two important factors regarding the prevalence of UTIs, as reported by Rahn [18]. In the current study, the isolates showed the highest resistance to NA (65.3%) and least resistance to CL (17.4%). Therefore, it appears that the use of nalidixic acid for the treatment of *E. coli* associated with UTIs in this center might be ineffective. However, since chloramphenicol is not routinely used clinically, aztreonam can still be a drug of choice for treating UTIs. Furthermore, the low resistance to chloramphenicol found in the study could be related to its low usage in routine treatments for UTIs.

Interestingly, most of the isolates were resistant to all the quinolone-containing antibiotics, (Table 2). This could be due to the reckless and inappropriate usage of the drugs [19]. Unfortunately, the rate of quinolone resistance in southwest Iran (Ahvaz) is significantly higher than other parts of Iran as reported by Pakzad et al. and even higher than what is found in other parts of the world [20]. According to the findings of this study, 64.07% of the isolates were ESBL producers. A lower rate of ESBL-producing strains has been reported in Japan and the USA [21, 22]. In Korea, it was reported to be 17.7% and 84% of ESBL-positive *E. coli* strains in 2003 and 2009, separately [15, 17]. Mood et al. reported that 42.5% of the *E. coli* isolates they studied were ESBL producers and this is lower than the finding of our study. The frequency of ESBL-producing *E. coli* was, therefore, higher in our study (except for the Korean study in 2009) than what has been reported from other countries and other Iranian metropolises [19]. Arbitrarily speaking, this high rate of ESBLs isolates in Iran and the lower frequency in some other countries could be attributed to several factors including but not limited to drug abuse pattern [23]. Studies have been conducted for *qnr* genes on *E. coli* strains in some Asian countries such as Korea (*qnr*B 5.6%), Japan (*qnr*A 6.5%), China (*qnr*A 8%) and Iran (*qnr*A 6%, *qnr*B 2.66%). Several pieces of research have been carried out in Europe (UK and Germany) and the USA on *qnr* genes among isolates of *E. coli* and other members of Enterobacteriaceae, but only Robicsec et al. reported *qnr*A (4%) in the USA [24, 25]. Moreover, the total percentage of *E. coli* isolates harboring *qnr* genes (59.88%) reported in this study, as shown in Table 3, is significantly higher in number and diversity than those reported in Europe, Asia, and the USA [23, 26, 27]. It seems there is a high frequency of transconjugation or transformation mechanisms of the genes occurring in southwestern Iran, and this could be attributed to the hot and humid climate [23] of southwest Iran. Fifty-eight percent of the strains harboring *qnr* genes were also ESBL producers (Table 3) and showed the highest level of resistance to quinolones (ESBL and *qnr* positive strains, as shown in Fig. 3). Moreover, the lowest level of resistance was seen among *qnr* and ESBL negative strains (Fig. 3). Contrary to our expectations, seven (4.19%) *qnr* and ESBL-negative isolates were resistant to all quinolones mentioned, which might be due to other different defensive mechanisms. On the other hand, nine (5.38%) *qnr* and ESBL-positive isolates were absolutely sensitive to all quinolones, most probably the resistance genes in those strains were silent [23, 28, 29]. Out of the total isolates harboring *qnr* genes, 10% (10/100) isolates contained *qnr*A, 21% (21/100) isolates contained *qnr*B, 41% (41/100) isolates contained *qnr*S, and 21%(21/100), 1% (1/100), 3% (3/100), and 3% (3/100) isolates contained a mix of *qnr*S-B, *qnr*B-A, *qnr*A-S, *qnr*A-B-S, respectively.

The *qnr*S gene seems to play a more significant role than *qnr*A and *qnr*B with regard to quinolones resistance since 65.85% (27/41) of the isolates harboring the *qnr*S gene were resistant to all quinolones (Table 4). As shown in Table 4, the combination of *qnr*S and B detected in some strain have increased the level of resistance to quinolone-containing antibiotics nearly to 100% (20 of 21), suggesting that the synergistic effects resulting from the combination of *qnr*S with *qnr*B in a strain may increase their defensive positions. Also, a significant correlation (*P* value < 0.05) was reported between *qnr*S gene and resistance to Cip and OF (Table 4). Attaining complete cure from UTIs caused by *E. coli* is highly challenging, and the presence of *qnr* genes and ESBLs production are said to be most responsible. However, some other factors such as the overexpression of efflux pump or declined intracellular concentration and mutations (in topoisomerase IV and DNA gyrase enzymes) could also be equally effective in this regard [12, 30, 31]. On the other hand, quinolones are the most prescribed antibiotics for UTI therapy in Iran. Today, one of the most important concerns is the risk of treatment failure among Iranian patients, especially those diagnosed with UTIs [20, 32]. To the best of our knowledge, this is the first study to investigate the presence of *qnr* genes (A, B, and S) in both ESBL-producing and non-ESBLs strains of *E. coli* isolated from patients diagnosed with UTIs and their resistance patterns to quinolones antibiotics in southwest Iran. Finally, the major limitations were small sample size, study of only seven drugs in terms of antimicrobial susceptibility, and single center-based design of this study.

## Conclusion

The frequency of *qnr* genes among clinical isolates of *E. coli* demonstrated in this study is higher than the usually reported frequency from other provinces of Iran. Resistance to quinolones antibiotics was highest among ESBL-producing isolates, especially those harboring *qnr*S among other determinants of *qnr* gene. There is a need for sensitive antibiotic stewardship especially in the health facilities of Ahvaz, Khuzestan province. Further research, with a wider scope, is needed to ascertain the gravity of quinolones resistance in Iran and to quickly act against its spread among other nosocomial pathogens.

## Abbreviations

AZT: Aztreonam
CAZ: Ceftazidime
CIP: Ciprofloxacin
CL: Chloramphenicol
CTX: Cefotaxime
DDST: Double disk synergism test
ESBL: Extended-spectrum β-lactamase
ICU: Intensive care unit
MIC: Minimum inhibitory concentration
NA: Nalidixic acid
No: Number
OF: Ofloxacin
OPD: Outpatient department
PMQR: Plasmid-mediated quinolone resistance
*qnr*: Quinolone resistance gene
R: Resistant
UTIs: Urinary tract infections

## References

[1] Jiang X, Yu T, Wu N, Meng H, Shi L (2014). Detection of *qnr*, aac (6′)-Ib-cr and qepA genes in Escherichia coli isolated from cooked meat products in Henan, China. Int J Food Microbiol.

[2] Rodríguez-Martínez JM, Cano ME, Velasco C, Martínez-Martínez L, Pascual Á (2011). Plasmid-mediated quinolone resistance: an update. J Infect Chemother.

[3] Jiang Y, Zhou Z, Qian Y, Wei Z, Yu Y, Hu S (2008). Plasmid-mediated quinolone resistance determinants *qnr* and aac (6′)-Ib-cr in extended-spectrum beta-lactamase-producing Escherichia coli and Klebsiella pneumoniae in China. J Antimicrob Chemother.

[4] Briales A, Rodriguez-Martinez JM, Velasco C, de Alba PD, Rodriguez-Bano J, Martinez-Martinez L (2012). Prevalence of plasmid-mediated quinolone resistance determinants *qnr* and aac (6′)-Ib-cr in Escherichia coli and Klebsiella pneumoniae producing extended-spectrum beta-lactamases in Spain. Int J Antimicrob Agents.

[5] Hopkins KL, Davies RH, Threlfall EJ (2005). Mechanisms of quinolone resistance in Escherichia coli and Salmonella: recent developments. Int J Antimicrob Agents.

[6] Yue L, Jiang HX, Liao XP, Liu JH, Li SJ, Chen XY (2008). Prevalence of plasmid-mediated quinolone resistance *qnr* genes in poultry and swine clinical isolates of Escherichia coli. Vet Microbiol.

[7] Ruiz E, Saenz Y, Zarazaga M, Rocha-Gracia R, Martinez-Martinez L, Arlet G (2012). *qnr*, aac (6′)-Ib-cr and qepA genes in Escherichia coli and Klebsiella spp.: genetic environments and plasmid and chromosomal location. J Antimicrob Chemother.

[8] Li X-Z (2005). Quinolone resistance in bacteria: emphasis on plasmid-mediated mechanisms. Int J Antimicrob Agents.

[9] Jeong J-Y, Yoon HJ, Kim ES, Lee Y, Choi S-H, Kim NJ (2005). Detection of *qnr* in clinical isolates of Escherichia coli from Korea. Antimicrob Agents Chemother.

[10] Wang A, Yang Y, Lu Q, Wang Y, Chen Y, Deng L (2008). Presence of *qnr* gene in Escherichia coli and Klebsiella pneumoniae resistant to ciprofloxacin isolated from pediatric patients in China. BMC Infect Dis.

[11] Wayne P. Clinical and laboratory standards institute. Performance standards for antimicrobial susceptibility testing 2016.

[12] Mohajeri P, Darfarin G, Farahani A. Genotyping of ESBL producing Uropathogenic Escherichia coli in west of Iran. Int J Microbiol. 2014;2014. https://www.hindawi.com/journals/ijmicro/2014/276941/. 10.1155/2014/276941 PMC4009276 24839441

[13] Pereira AS, Andrade SS, Monteiro J, Sader HS, Pignatari AC, Gales AC (2007). Evaluation of the susceptibility profiles, genetic similarity and presence of *qnr* gene in Escherichia coli resistant to ciprofloxacin isolated in Brazilian hospitals. Braz J Infect Dis.

[14] Cattoir V, Poirel L, Rotimi V, Soussy CJ, Nordmann P (2007). Multiplex PCR for detection of plasmid-mediated quinolone resistance *qnr* genes in ESBL-producing enterobacterial isolates. J Antimicrob Chemother.

[15] Park JH, Lee SH, Jeong SH, Kim BN, Kim KB, Yoon JD (2003). Characterization and prevalence of Escherichia coli and Klebsiella pneumoniae isolates producing an extended-spectrum beta-lactamase from Korean hospitals. Korean J Lab Med.

[16] Jones RN, Pfaller MA, Doern GV, Erwin ME, Hollis RJ, Group CS (1998). Antimicrobial activity and spectrum investigation of eight broad-spectrum β-lactam drugs: a 1997 surveillance trial in 102 medical centers in the United States. Diagn Microbiol Infect Dis.

[17] Park Y, Kang H-K, Bae IK, Kim J, Kim J-S, Uh Y (2009). Prevalence of the extended-spectrum β-lactamase and *qnr* genes in clinical isolates of Escherichia coli. Korean J Lab Med..

[18] Rahn DD (2008). Urinary tract infections: contemporary management. Urol Nurs.

[19] Mood EH, Meshkat Z, Izadi N, Rezaei M, Jamehdar SA, Nasab MN. Prevalence of quinolone resistance genes among extended-spectrum B-lactamase-producing Escherichia coli in Mashhad, Iran. Jundishapur J Microbiol. 2015;8(12). 10.5812/jjm.16217 PMC4746706 26870307

[20] Pakzad I, Ghafourian S, Taherikalani M (2011). *Qnr* prevalence in extended-spectrum beta-lactamases (ESBLs) and none-ESBLs producing Escherichia coli isolated from urinary tract infections in central of Iran. Iran J Basic Med Sci.

[21] Munshi M, Haider K, Rahaman M, Sack D, Ahmed Z, Morshed M (1987). Plasmid-mediated resistance to nalidixic acid in Shigella dysenteriae type 1. Lancet.

[22] Martínez-Martínez L, Pascual A, Jacoby GA (1998). Quinolone resistance from a transferable plasmid. Lancet.

[23] Corkill JE, Anson JJ, Hart CA (2005). High prevalence of the plasmid-mediated quinolone resistance determinant *qnr*A in multidrug-resistant Enterobacteriaceae from blood cultures in Liverpool, UK. J Antimicrob Chemother.

[24] Poirel L, Leviandier C, Nordmann P (2006). Prevalence and genetic analysis of plasmid-mediated quinolone resistance determinants *Qnr*A and *Qnr*S in Enterobacteriaceae isolates from a French university hospital. Antimicrob Agents Chemother.

[25] Allou N, Cambau E, Massias L, Chau F, Fantin B (2009). Impact of low-level resistance to fluoroquinolones due to *qnr*A1 and *qnr*S1 genes or a *gyr*A mutation on ciprofloxacin bactericidal activity in a murine model of Escherichia coli urinary tract infection. Antimicrob Agents Chemother.

[26] Jonas D, Biehler K, Hartung D, Spitzmüller B, Daschner FD (2005). Plasmid-mediated quinolone resistance in isolates obtained in German intensive care units. Antimicrob Agents Chemother.

[27] Robicsek A, Strahilevitz J, Sahm D, Jacoby G, Hooper D (2006). *qnr* prevalence in ceftazidime-resistant Enterobacteriaceae isolates from the United States. Antimicrob Agents Chemother.

[28] Xue Y, Chen J, Hua Y. Resistance of strains producing extended-spectrum [beta]-lactamases and genotype distribution among Escherichia coli in China. Pak J Zool. 2012;44(2). http://zsp.com.pk/vol-44%5B2%5D.html, http://zsp.com.pk/pdf44/457-461%20_23_%20PJZ-725-11.pdf.

[29] Vasilaki O, Ntokou E, Ikonomidis A, Sofianou D, Frantzidou F, Alexiou-Daniel S (2008). Emergence of the plasmid-mediated quinolone resistance gene *qnr*S1 in Escherichia coli isolates in Greece. Antimicrob Agents Chemother.

[30] Oktem IMA, Gulay Z, Biçmen M, Gur D, Group HPS (2008). *qnr*A prevalence in extended-spectrum beta-lactamase-positive Enterobacteriaceae isolates from Turkey. Jpn J Infect Dis.

[31] Peymani A, Farivar TN, Najafipour R, Mansouri S (2016). High prevalence of plasmid-mediated quinolone resistance determinants in Enterobacter cloacae isolated from hospitals of the Qazvin, Alborz, and Tehran provinces, Iran. Rev Soc Bras Med Trop.

[32] Firoozeh F, Zibaei M, Soleimani-Asl Y (2014). Detection of plasmid-mediated *qnr* genes among the quinolone-resistant Escherichia coli isolates in Iran. J Infect Dev Ctries.

